# Is nail-plate docking worth the effort? A biomechanical analysis of docking a plate and a nail in peri-implant femur fractures

**DOI:** 10.3389/fbioe.2024.1392631

**Published:** 2024-06-04

**Authors:** Justus Bremer, Maximilian Heilig, Philipp Heilig, Stefanie Hölscher-Doht, Rainer H. Meffert, Martin C. Jordan

**Affiliations:** ^1^ Department of Orthopedic Trauma, Hand, Plastic and Reconstructive Surgery, University Hospital Würzburg, Würzburg, Germany; ^2^ Center for Orthopaedics, Trauma Surgery and Rehabilitation Medicine, University Medicine Greifswald, Greifswald, Germany

**Keywords:** proximal femur fracture, proximal femoral nail, implant docking, cephalomedullary nail, peri-implant femur fracture, femur plate, nail-plate docking

## Abstract

**Purpose:**

The ideal treatment of peri-implant femur fractures (PIFFs) remains unclear due to the thin clinical and biomechanical evidence concerning the most suitable form of osteosynthesis. The purpose of the present study was thus to determine the biomechanical stability that results from combining a cephalomedullary nail and a plate for proximal PIFFs, especially when the nail–plate docking technique is applied.

**Methods:**

Twenty four PIFFs were simulated in both 12 foam and 12 composite specimens and were stabilized via a combination of a cephalomedullary nail and a plate. The control group (*n* = 6) had a nail and a plate without a connection, while the intervention group (*n* = 6) had a screw that connected the plate with the interlocking screw hole of the nail, thereby creating a nail–plate docking system. The specimens were evaluated under axial and torsional loading using a material-testing machine and a 3D metrology system.

**Results:**

The data regarding stiffness, failure load, and failure displacement showed significantly higher stability for specimens without nail–plate docking. For docked specimens, a non-significant trend toward a higher resistance to torque was observed. Both techniques displayed no significant difference in fracture gap displacement or total displacement.

**Conclusion:**

The present study suggests that nail–plate docking of a cephalomedullary nail, and a plate significantly decreases the stiffness and stability of osteosynthesis under axial loading. However, there seems to be a tendency toward higher resistance to torque. Therefore, surgeons should consider this technique if higher torsional stability is necessary, and they should decide against it, if axial stability is preferred.

## 1 Introduction

Hip fractures frequently occur in the elderly. They are often caused by minor falls in the presence of osteoporosis and occur more frequently in aging populations in several countries (Bergh et al., 2020; Rupp et al., 2021). In general, hip fractures are treated via surgical intervention using either osteosynthesis or prosthetic replacement. In such treatment, osteosynthesis is associated with a significant risk of peri-implant femur fracture (PIFF). PIFFs represent a distinct clinical entity from the better-known peri-prosthetic femur fracture (PPFF) and thus require different surgical considerations. The ever-increasing number of hip fractures is also resulting in similarly increasing numbers of PIFFs (Robinson et al., 2002; Norris et al., 2012; Chan et al., 2018; Kruse et al., 2022) and is thus becoming a severe orthopedic challenge. PIFFs have a comparable cumulative incidence to that of PPFFs and are associated with high mortality and complication rates (Lang et al., 2017; Kruse et al., 2022). Moreover, the treatment for PIFFs is demanding because standardized surgical procedures are not transferable. A broad variety of pre-existing implants, different fracture morphologies, and poor bone quality are only some of the problems encountered [8, 9].

**FIGURE 1:**
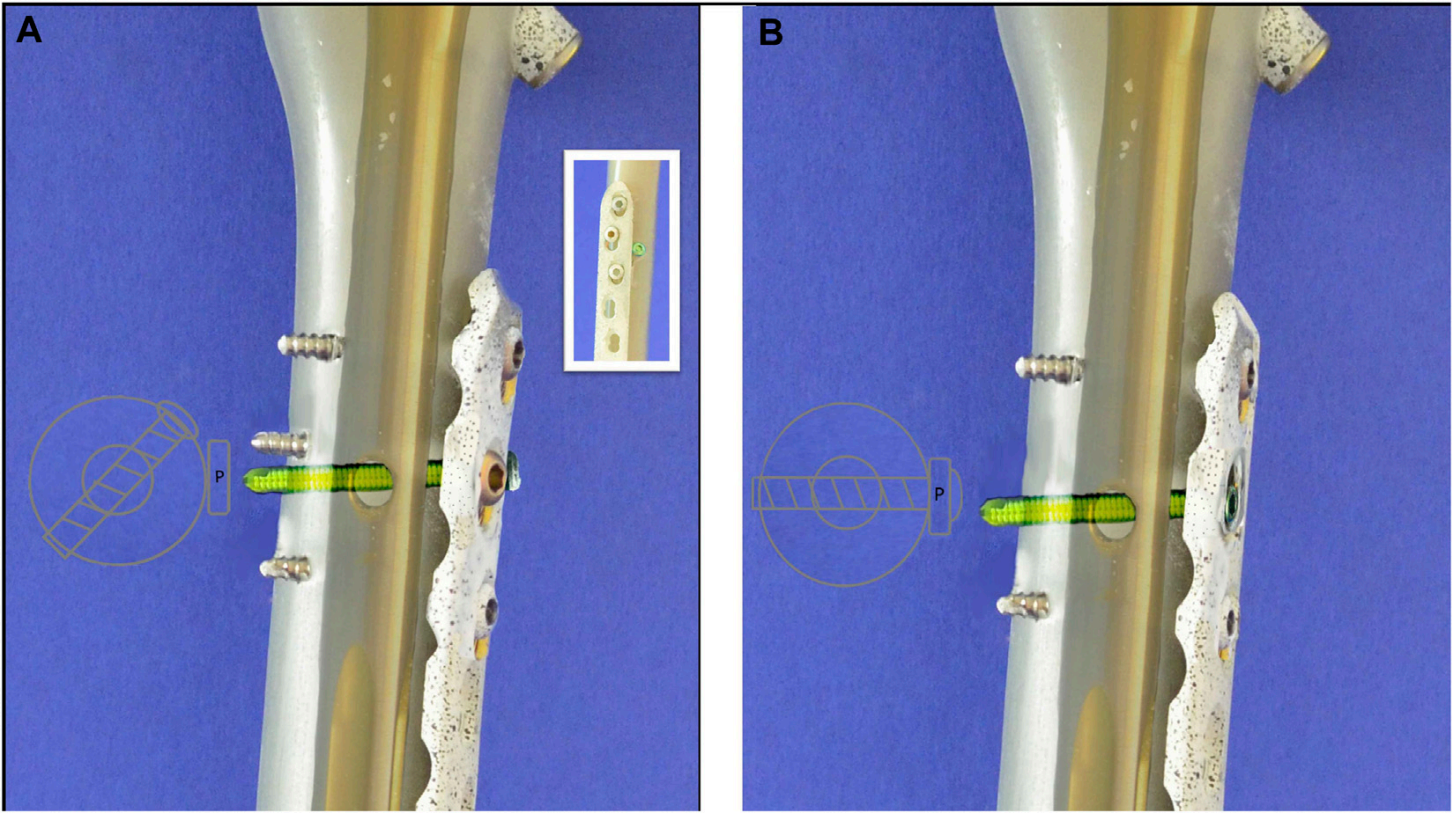
Schematic Illustraion of the different treatments: **(A)** Demonstration of the distal interlocking screw (green) separately from the plate (non-docking) with a sketch of the screw’s trajectory from above. **(B)** Demonstration of the locking screw (green) connecting nail and plate, creating a nail-plate docking system with a sketch of the screw’s trajectory from above.

The complexity of PIFFs is underlined by the struggle to implement an internationally accepted classification system [6, 10, 11]. Furthermore, literature on PIFFs is rare, and the biomechanical understanding of the phenomenon remains limited. In most cases, PIFFs occur around a pre-existing nail or plate, and a treatment strategy might require a combination of a nail with a plate. Usually, the two implants lack a link to each other (Figure 1A); however, it is also possible to connect them using a screw that runs through the hole of the distal interlocking screw of the cephalomedullary nail and the screw hole of the plate, thereby creating a nail–plate docking system (Figure 1B; Takai et al., 2022). Currently, the biomechanical effect of the nail–plate docking technique is unclear.

The present study thus aimed to evaluate the difference in stability between a cephalomedullary nail with a docked plate and a non-docked control group from a biomechanical perspective. In order to compare the techniques, two biomechanical test setups were implemented that applied axial loading and torque under cyclic testing. Therefore, the present study adds to the existing biomechanical evidence and can aid surgeons in a more informed decision-making process.

## 2 Methods

### 2.1 Specimen preparation and biomechanical testing

**FIGURE 2:**
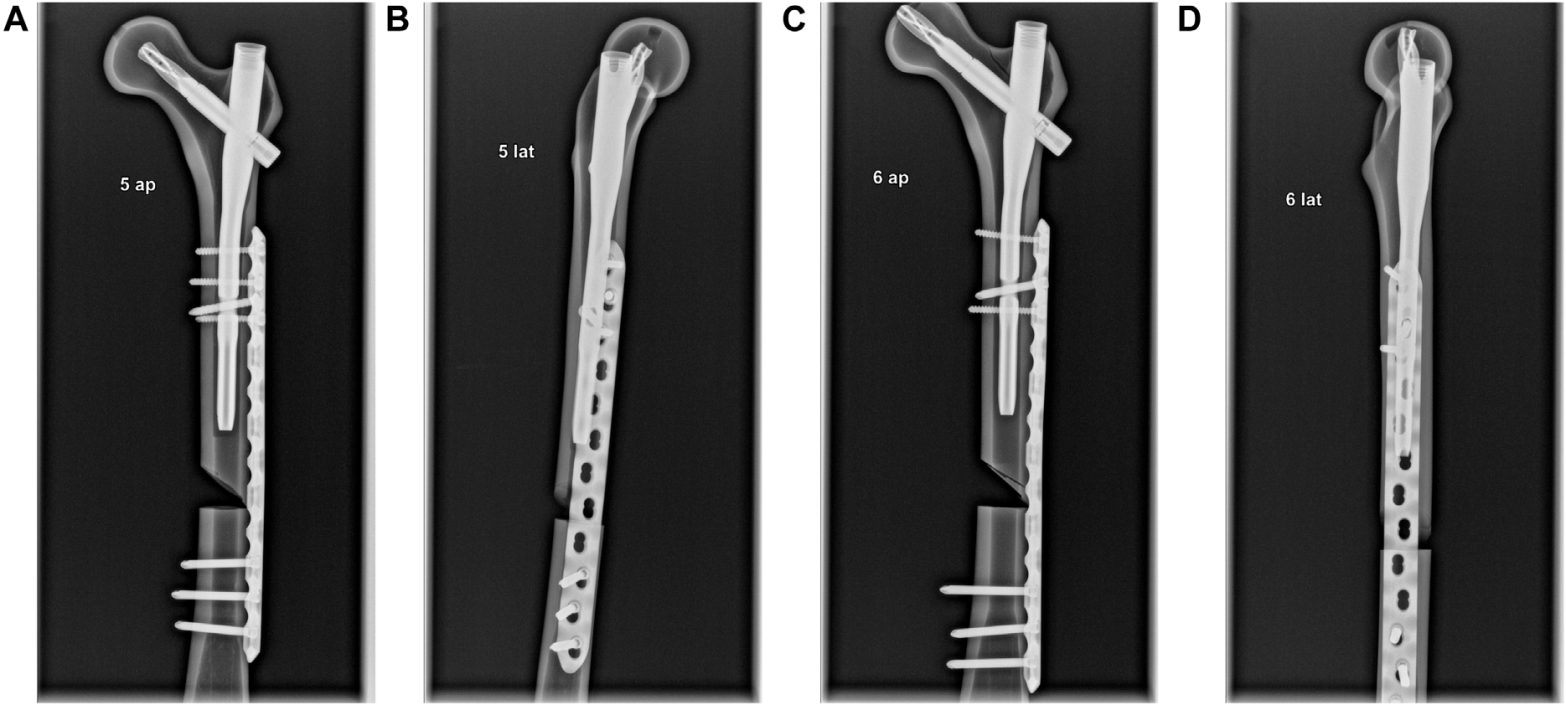
Radiographs of two differently treated specimens in a.p. and lateral view. **(A)** Ap. view. Three anterior screws and one distal interlocking screw in the proximal part of the plate indicate a non-docked specimen. **(B)** Lateral view. Anterior plate positioning–caused by the nail’s distal interlocking screw–is visible. **(C)** Ap. view. Three screws indicate a docked specimen. **(D)** Lateral view. Central plate positioning is visible.

**FIGURE 3:**
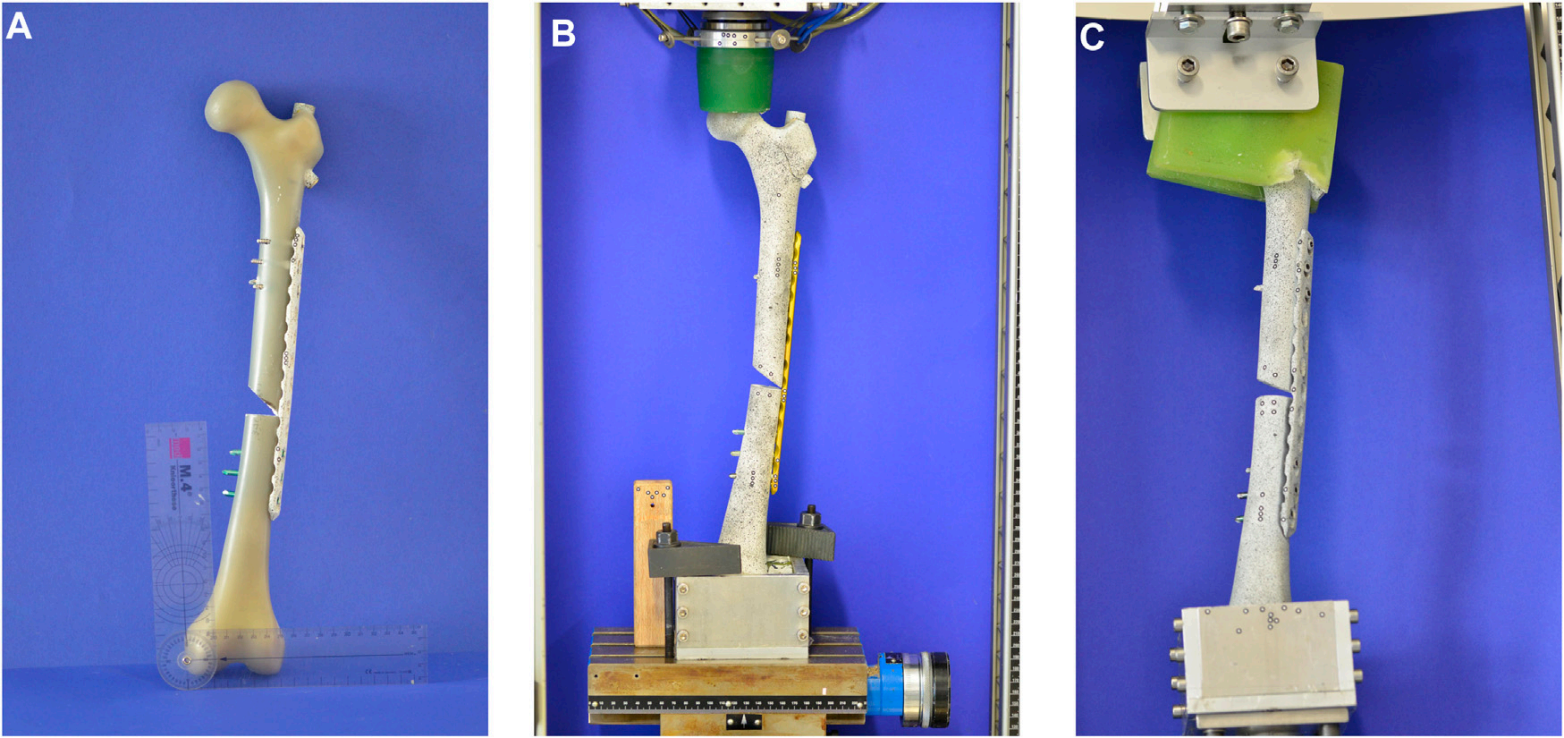
The pictures show the specimens and the custom-made test setups for axial and torsional loading. **(A)** A 4th Gen. composite femur treated with a PFNA and a broad LCP plate. **(B)** Custom-made test setup for axial loading. The pressure shell transferred axial loading to the specimen. **(C)** Custom-made test setup for torsional loading. The impression molds were rigidly clamped to the specimen and therefore enabled torsional forces to be transferred.

Specimen preparation followed a standardized protocol. For the pre-series, right solid-foam femora with a 9.5 mm canal were used (Synbone AG; Zizers, Schweiz; Model LD2162; polyurethane foam), which are referred to in the present text as “foam specimens.” After establishing a preparation and testing routine, 4^th^ Gen. left composite femora with seventeen pounds per cubic foot (PCF) of solid-foam filling and a 10 mm canal (Model No. 3403-104, Sawbones, Vashon, WA, United States) were chosen for further testing (Figure 3A), which are referred to in the present text as “composite specimens.” According to the manufacturer, seventeen PCF corresponds to a bone density of between 240 and 320 mg/cm^3^. Each specimen was treated with a 200 mm × 10 mm Proximal Femoral Nail Antirotation™ and a 100 mm helical blade (DePuy Synthes, Johnson & Johnson Medical GmbH, Umkirch, Germany). Next, a broad LCP plate (224 mm length, 12 hole, DePuy Synthes, Johnson & Johnson Medical GmbH, Umkirch, Germany) was attached on the lateral anterior side of the specimen. In the control group, three proximal cortical screws and three distal locking screws were used to fixate the plate. In the intervention group, a locking screw in the proximal part of the plate served simultaneously as a static interlocking screw for the nail. Afterward, a wedge fracture was created in the middle third of the shaft using an oscillating saw. Correct positioning of all implants and screws was verified and documented via X-ray fluoroscopy (Figure 2).

Biomechanical testing was conducted using two custom-made test setups and a static material Testing Machine (Zwick Z020, ZwickRoell, Ulm, Germany). For cyclic loading, the test setup consisted of an embedding form and a pressure shell made from polymethylmethacrylate. The pressure shell was curved inward in order to simulate axial loading from the hip joint and was placed on a bearing in order to account for non-axial forces in the *x*/*z*-direction (Figure 3B). Foam specimens were tested for 3,000 cycles ranging from 100 to 400 N, and composite specimens were tested for 3,000 cycles ranging from 100 to 600 N. For the composite specimens, cyclic loading was followed by a load-to-failure test. In order to allow for torsional loading, the pressure shell was replaced with impression molds made of polymethylmethacrylate from the specimens (Figure 3C). A torque of 10 Nm was applied for 600 cycles in a counter-clockwise direction under simultaneous axial loading of 200 N before a torsional load-to-failure test was conducted. Loading cycles were applied at a rate of 0.25 Hz. The number of tested specimens per group was four for axial loading and two for torsional loading. The testXpertII software (Version 3.6, ZwickRoell, Ulm, Germany) recorded torque (Nm), displacement (mm), and load (N) during testing at a rate of 100 Hz and sketched the data in a load-displacement graph. Stiffness was determined as the gradient in a force-strain-graph and calculated based on sensor data by dividing the difference in force and displacement at the loading point and the relief point. Technical specifications of the used load cell can be taken from Supplementary Table 1.

**FIGURE 4:**
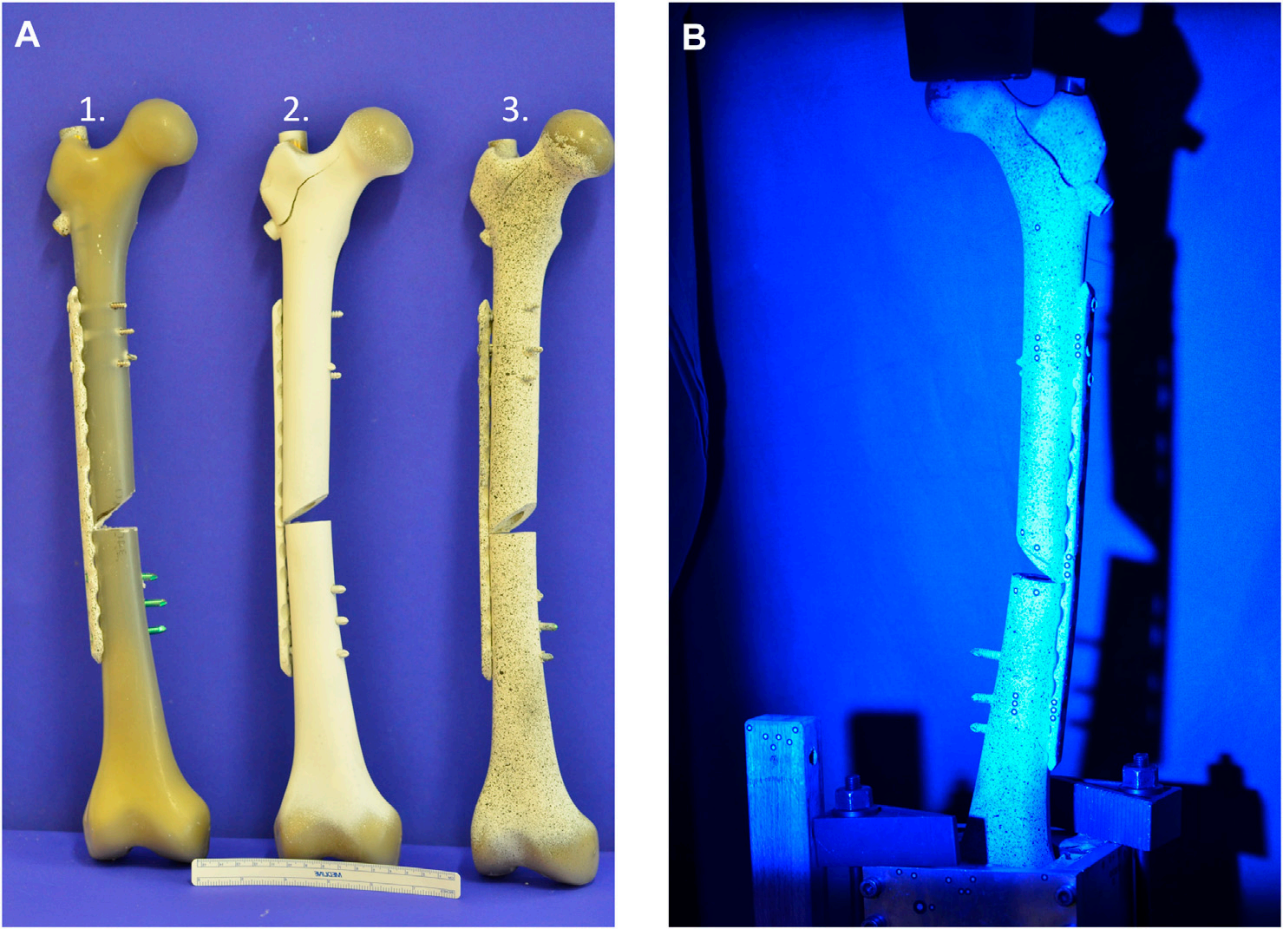
The pictures show the composite specimens in preparation for the 3D metrology system. **(A)** 1. Composite specimen without pattern. 2. Composite specimen with background coting 3. Composite specimen with stochastic pattern. **(B)** Composite with pattern during optical measurement.

Moreover, a stochastic pattern and reference markers with a diameter of 1.5 mm were attached to the surface of each specimen (Figure 4A). This process allowed optical measurements (Figure 4B) with a 3D metrology system (ARAMIS 3D Professional, Carl Zeiss GOM Metrology GmbH, Braunschweig, Germany). The 3D metrology system consisted of a sensor with two 6 MP cameras for optical measurements and was connected to the material-testing machine in order to allow the measurements to be matched with the current loading. The reference markers were subdivided into a proximal, medial, and distal component. Calibration was performed before each test, and accuracy was confirmed (0.3–0.03 mm). Concerning the different reference markers, those on either side of the fracture gap (medial component) were considered the most significant and were used to determine fracture gap displacement during axial loading. The metrology system measured all setting cycles and every 10th measuring cycle (see Supplementary Figure 1).

Statistical analysis was performed using SPSS V.28 (IBM, NY, United States). In the first step, explorative data analysis was used to calculate mean values and standard deviations. Next, normal distribution was verified using the Shapiro–Wilk test. Additionally, the corresponding Q–Q plots were evaluated. If normal distribution was given, Leven’s test decided whether normal or Welch ANOVA was necessary for analysis between groups. For normal ANOVA, Tukey’s *post hoc* test was used for further analysis. If Welch ANOVA was used, the Games–Howell test was performed. If normal distribution was not given, the Kruskal–Wallis and Mann–Whitney U tests were used for further analyses. When only two groups were compared with each other, either the *t*-test or the Kruskal–Wallis test was used depending on the normal distribution. The level of significance was set to *p* < 0.05 for all statistical analyses.

## 3 Result

### 3.1 Total displacement and stiffness

**FIGURE 5:**
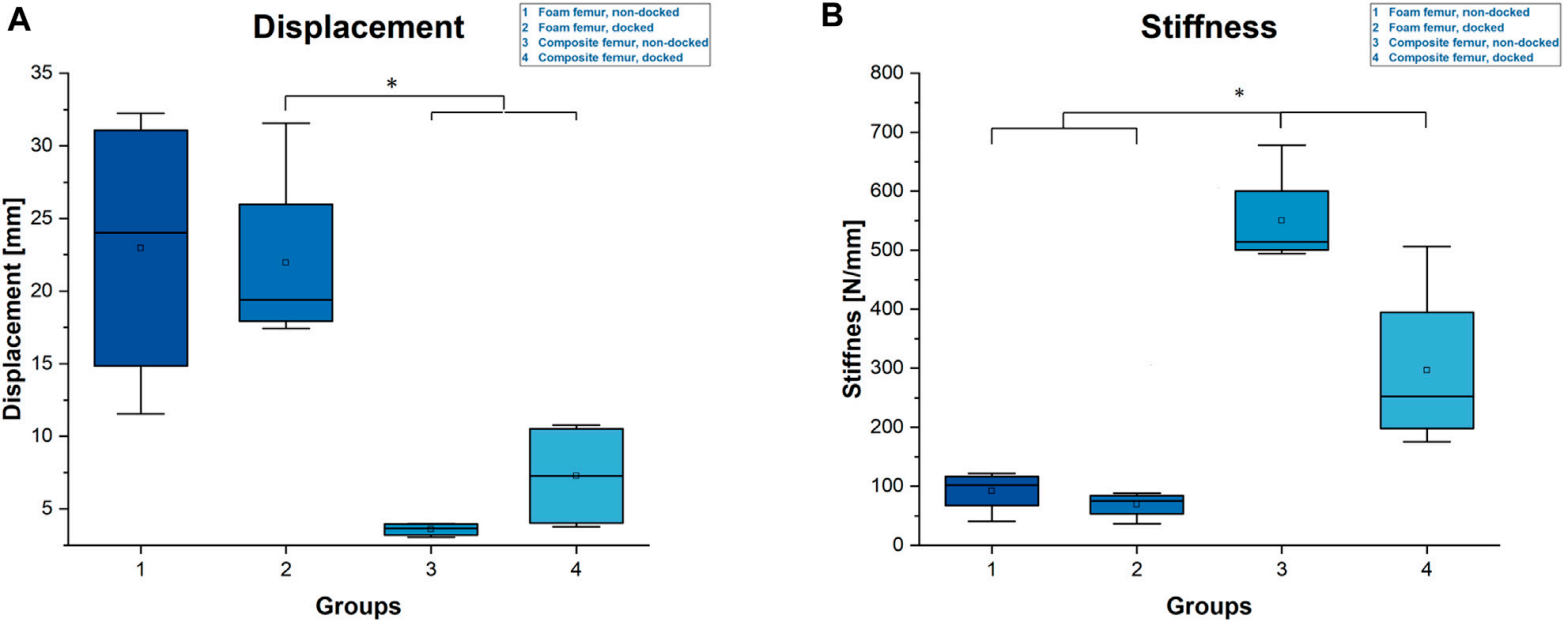
Diagrams of total displacement and stiffness. **(A)** Total displacement. Statistical differences are marked with an asterisk (*). **(B)** Calculated stiffness during cyclic loading. Statistical differences are marked with an asterisk (*).

Total displacement after cyclic loading was 23 ± 4.9 mm for the non-docked foam specimens and 22 ± 6.5 mm for the docked foam specimens. The non-docked composite specimens averaged at 3.6 ± 0.5 mm, and the nail–plate docked specimens averaged at 7.3 ± 3.8 mm. The differences within all four groups were not significant; however, both composite groups individually showed a significantly lower displacement to the average for the docked foam specimens (Figure 5A).

Regarding stiffness, the non-docked composite specimens showed the highest mean value, with 550 ± 86 N/mm, which was followed by the docked composite specimens, with an average stiffness of 296 ± 146 N/mm. The mean stiffness of the non-docked foam specimens was 91 ± 35 N/mm, while the mean stiffness of the docked foam specimens was 68 ± 23 N/mm. While the docked foam specimens were not significantly less stiff than the non-docked foam specimens, the composite specimens were significantly stiffer than the foam specimens, and the difference in stiffness between the composite specimens was significant (Figure 5B).

### 3.2 Failure load and failure displacement

**FIGURE 6:**
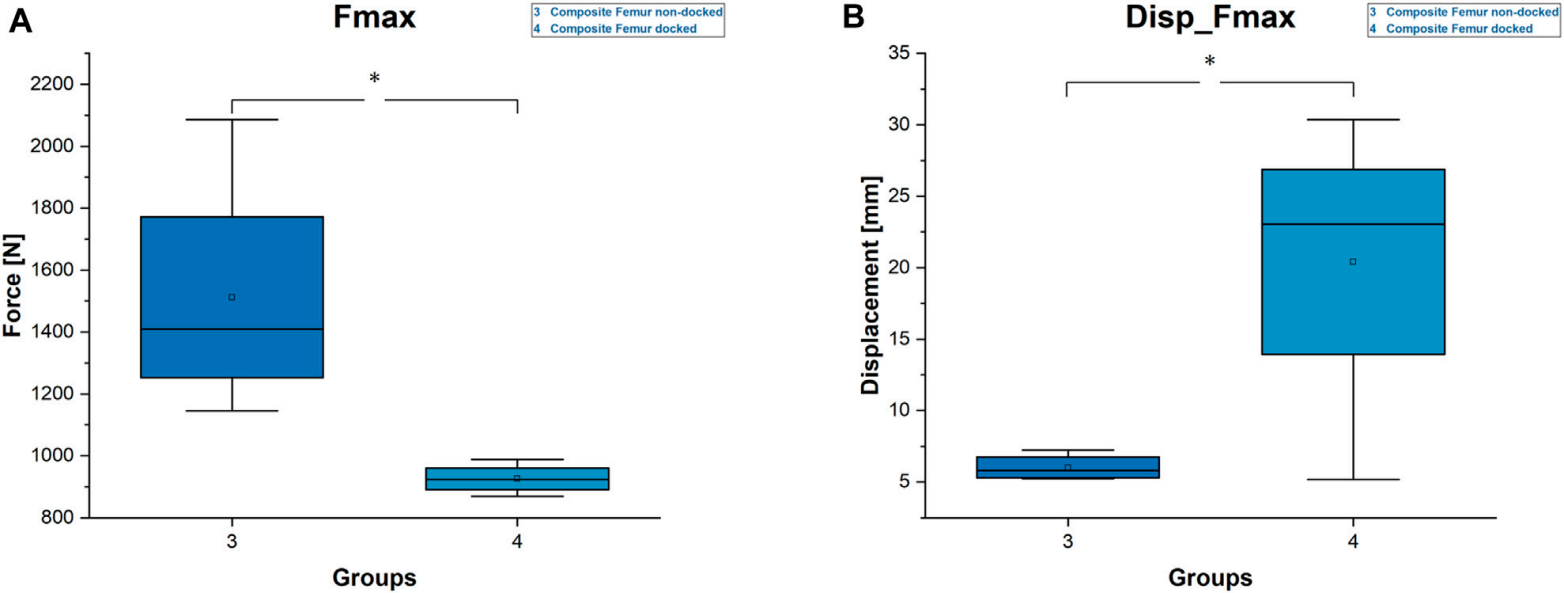
Diagrams of Fmax and displacement at Fmax. **(A)** Fmax. Statistical differences are marked with an asterisk (*). **(B)** Displacement Fmax. Statistical differences are marked with an asterisk (*).

The mean failure load (F_max_) was highest for the non-docked specimens, with 1,512 ± 404 N, followed by 926 ± 48 N for the docked composite specimens (Figure 6A). The displacement at the point of failure (Disp_F_max_) displayed a corresponding trend: The mean displacement was lowest for the non-docked specimens, with 6 ± 0.9 mm, followed by 20.4 ± 10.7 mm for the docked specimens. The differences were statistically significant for both parameters (Figure 6B).

### 3.3 Displacement fracture gap and maximum torque

**FIGURE 7:**
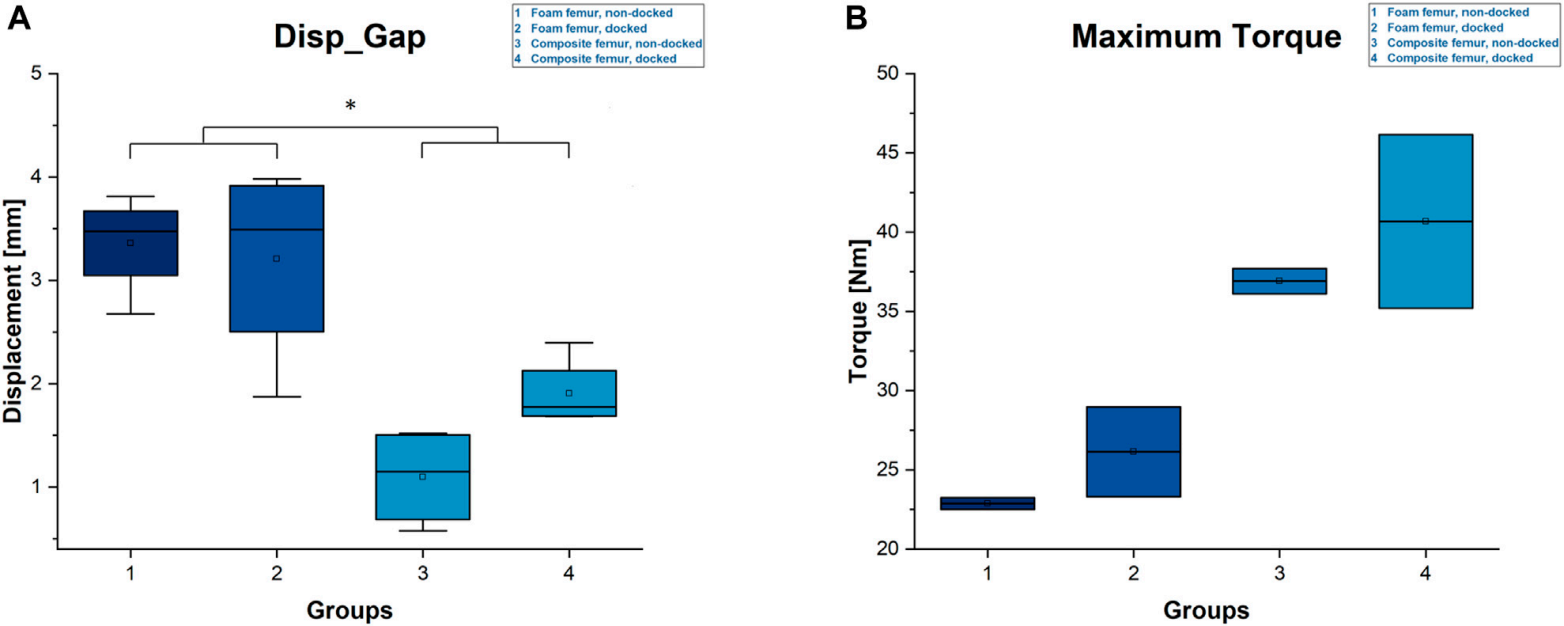
Diagrams of displacement fracture gap and maximal torque. **(A)** Displacement fracture gap. Statistical differences are marked with an asterisk (*). **(B)** Maximum torque.

In addition to the parameters outlined above, the closing of the fracture gap during cyclic loading was measured optically. The fracture gap closed by 1.1 ± 0.5 mm for the non-docked specimens and by 1.9 ± 0.3 mm for the docked composite specimens. These values were higher in the non-docked and docked foam specimens, with 3.4 ± 0.5 mm and 3.2 ± 1 mm, respectively (Figure 7A). Both the docked and non-docked composites had significantly less displacement than the foam femurs.

The average maximum torque achieved in the torsional load-to-failure testing was 22.9 ± 0.4 Nm for the non-docked foam specimens and 26.1 ± 2.8 Nm for the docked foam specimens. The non-docked composite specimens reached an average of 36.9 ± 1.1 Nm, while the docked composite specimens achieved 40.7 ± 7.7 Nm on average (Figure 7B).

## 4 Discussion

PIFFs are a rare yet severe complication that arises after nailing a proximal femur fracture. While clinical evidence has become more solid over the last few years, with attempts having been made to classify the fractures (Chan et al., 2018; Egol et al., 2019; Videla-Ces et al., 2019) and to establish a treating algorithm (Bidolegui et al., 2023), the general evidence on PIFFs is rare and has focused on the risk of secondary fracture rather than on treatment (Daner et al., 2017; Hamandi et al., 2020; Breceda et al., 2021; Saggi et al., 2022). To our knowledge, no biomechanical study has thus far investigated the treatment of PIFFs with nail–plate docking.

Our results lead to the assumption that docking a cephalomedullary nail with a plate does not improve the axial stability of the construct. This assumption is supported by the significantly higher stiffness and maximum load as well as by the lower displacements for the non-docked specimens. Contrary to the hypothesized expectation, nail–plate docking seems to have lowered biomechanical stability. This finding could be due to the positioning of the plate in the non-docked specimens. For such specimens, the plate had to be positioned more anteriorly in order to not interfere with the PFNA distal interlocking screw. For docked specimens, plate positioning was dictated by the interlocking screw of the cephalomedullary nail and therefore had to be more central (Figure 2). The more anterior positioning of the plate led to significantly greater stiffness by acting as an axial strut against the bending movement of the femur. This finding was supported by the statistically significant differences for stiffness, maximum force, and displacement at maximum force in non-docked specimens (Figures 5B, 6). Furthermore, both the total displacement and the displacement of the fracture gap did not yield significant differences for either treatment, which supported the thesis that the additional effort needed for docking is questionable (Figures 5A, 7A). However, there was a trend toward higher torsional stability in docked systems. This finding indicates that nail–plate docking could lead to better resistance against torque. It is noteworthy that long bones had poor resistance against torque, though this finding requires further evaluation.

**FIGURE 8:**
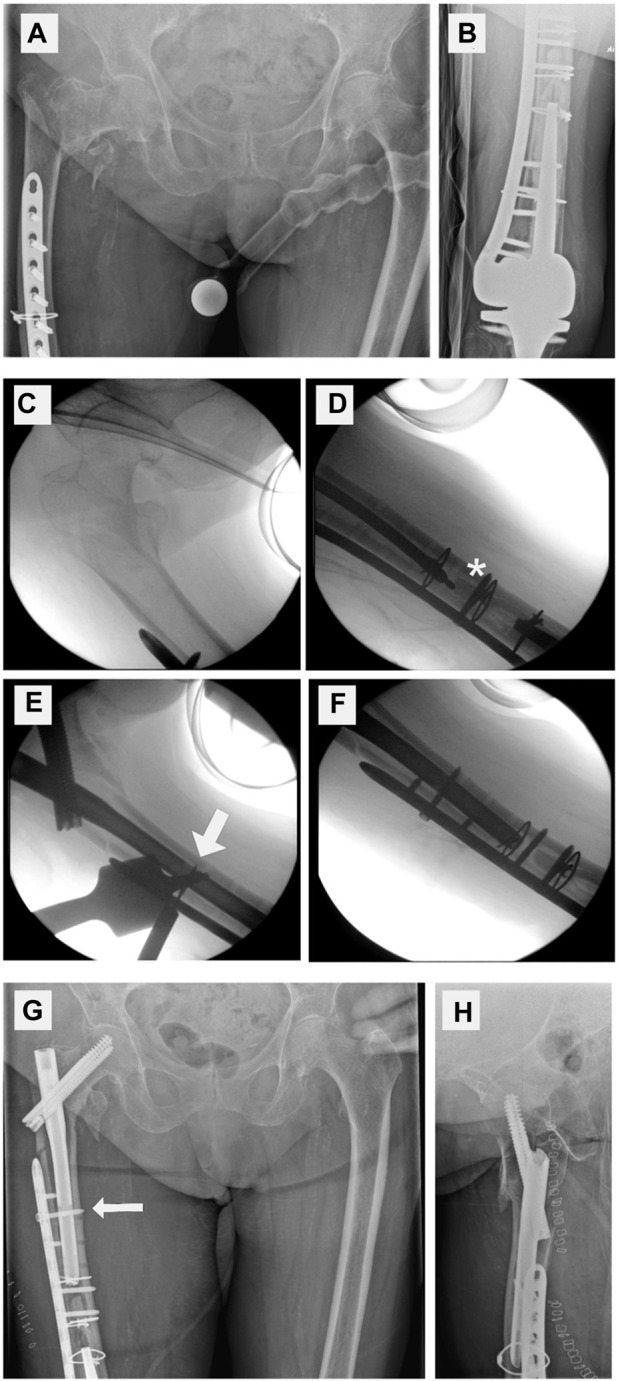
Clinical case with nail–plate docking technique. **(A)** Proximal femur fracture. **(B)** Distal locking plate. **(C–F)** Intraoperative fluoroscopy. Closed reduction, reaming up to bone cement (*), drilling through a plate hole and the nail (broad arrow), and placing a docking screw. Postoperative X-ray **(G,H)** showing the docking screw (thin arrow).

**FIGURE 9:**
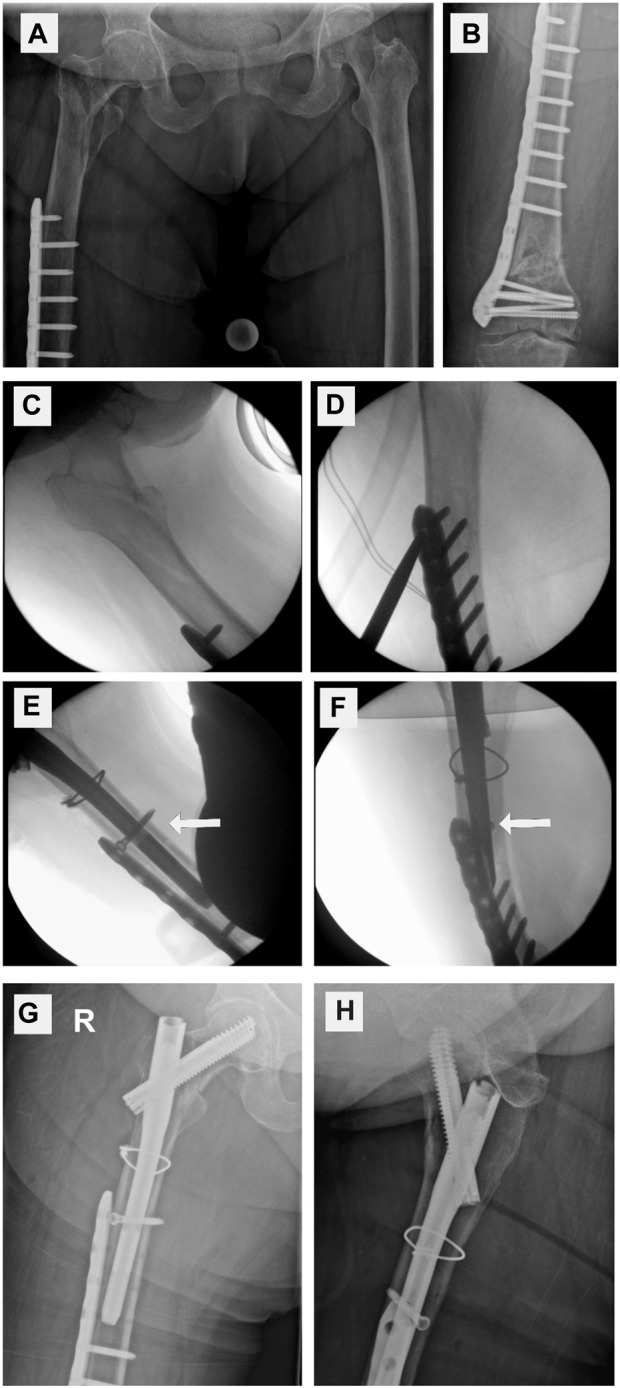
Clinical case without implant docking. **(A,B)** Proximal peri-implant femur fracture next to an existing distal femur plate **(C–F)** indicates the removal of several screws that keep the plate in place. The white arrow indicates a distal interlocking screw that is outside the plate, thereby showing no docking. **(G–H)** Postoperative X-ray of fracture fixation without nail–plate docking technique.

The treatment of a PIFF with a nail–plate docking system is a rather difficult operation depending on whether the plate or the nail is pre-existing. Either way, using one screw to connect a plate and a nail is troublesome. Although the process is feasible in some cases (Figure 8), it is associated with both a higher exposure to radiation and a prolonged operative time, the latter of which increases the risk of infection and the use of anesthesia. Normal implant placement without docking is an easier procedure that requires less extensive radiological scanning for screw placement (Cheng et al., 2017; Cheng et al., 2018; McGain et al., 2020). Regarding the ideal technique, the use of polyaxially locking plates compared with monoaxially locking plates is time-saving, as is the use of a dynamic hole of distal interlocking screw rather than a static hole of distal interlocking screw, the latter of which has been reported by Takai et al. (Hanschen et al., 2014; Takai et al., 2022). In clinical practice, changing the nail to a longer implant instead of plating is preferred by some surgeons; however, Goodnough et al. revealed that revision to a longer nail instead of plating is associated with increased mortality (Goodnough et al., 2021). By extension, two cases of clinical hip fracture with a pre-existing plate in which subsequent nailing was mandatory are outlined below in order to highlight the fact that nail–plate docking is possible in a different sequence (Figures 8, 9).

The present study is not without limitations. First, our test setup was limited to a PIFF at the tip of a pre-existing cephalomedullary nail in a formally healed inter-trochanteric fracture (Chan N1A) (Chan et al., 2018). Second, the results could have been different if the distal interlocking screw had been placed in the dynamic position of distal interlocking screw hole. Third, the number of tested specimens was low, and an additional study with a higher number of specimens might thus be necessary to confirm our findings. Additionally, plates used for PIFF treatment usually cover most of the femoral length and the distal screw is further away from the fracture.

In their biomechanical study, Harris et al. demonstrated the importance of implant overlap in PIFFs. Kissing or overlapping instrumentation increases load to failure and creates a more biomechanically stable construct. Femora with non-instrumented osseous intervals should be avoided (Harris et al., 2003). Our findings demonstrate the effect of docking in addition to overlapping, thereby enabling surgeons to make more informed decisions when treating patients with such complex fractures. Future implant development should thus consider peri-implant fracture management and allow easy docking and overlapping options in order to improve stability.

## 5 Conclusion

The present study suggests that docking a cephalomedullary nail and a plate significantly decreases stiffness and stability under axial loading. However, there seems to be a tendency toward higher stability to torque. Therefore, surgeons should consider nail–plate docking if higher stability to torque is necessary, and they should decide against such a procedure if axial stability is preferred. Either way, the biomechanical data indicate that the docking technique does not yield any significant effect, and the effort of conducting the implant link during surgery thus might not be justified.

## Data Availability

The raw data supporting the conclusions of this article will be made available by the authors, without undue reservation.

## Supplementary material

SUPPLEMENTARY FIGURE S1 Optical recording of the metrology system and the corresponding graph. **(A)** High-resolution 267 image of the evaluated fracture gap movement. **(B)** Graph of the recorded data during cyclic 268 testing.
