# Supplementary figures and images for: Is nail-plate docking worth the effort? A biomechanical analysis of docking a plate and a nail in peri-implant femur fractures
Source: Front Bioeng Biotechnol. 2024 Jun 4;12:1392631. doi: 10.3389/fbioe.2024.1392631 (PMC11184161; doi:10.3389/fbioe.2024.1392631)

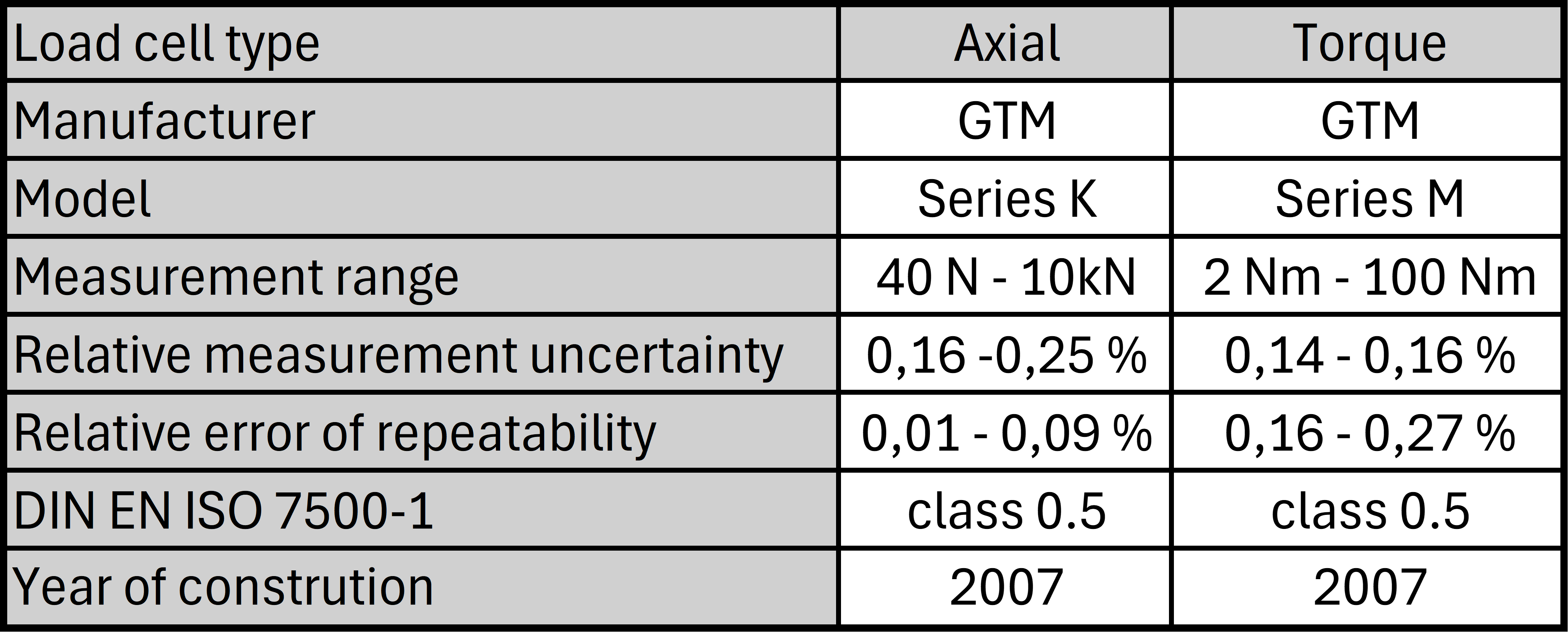

Supplement: Supplementary file 1 [file Image2.TIF]

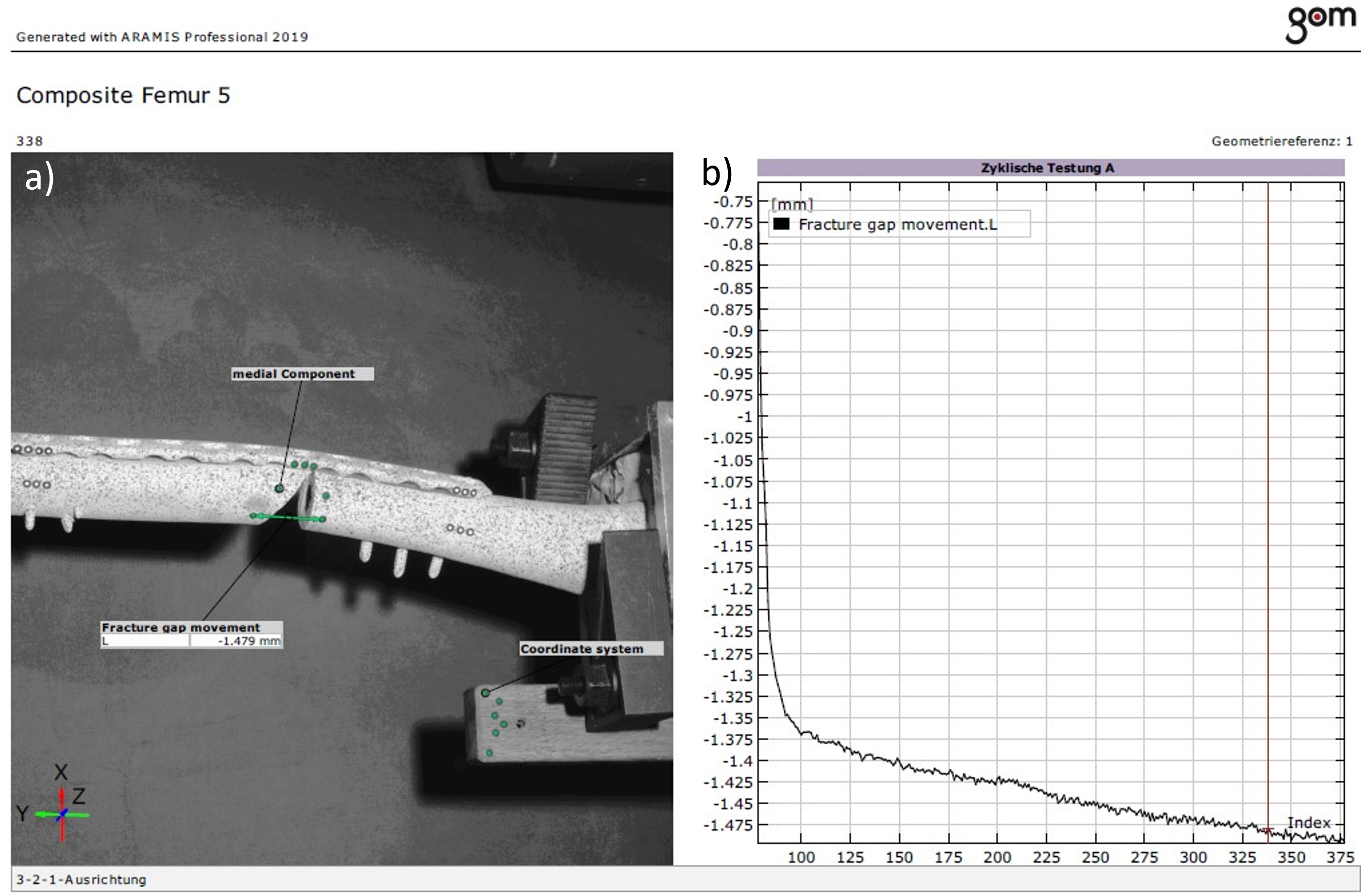

Supplement: Supplementary file 2 [file Image1.TIF]
